# Animal Health Management Practices Among Smallholder Livestock Producers in Australia and Their Contribution to the Surveillance System

**DOI:** 10.3389/fvets.2019.00191

**Published:** 2019-06-18

**Authors:** Marta Hernández-Jover, Lynne Hayes, Robert Woodgate, Luzia Rast, Jenny-Ann L. M. L. Toribio

**Affiliations:** ^1^Graham Centre for Agricultural Innovation (An Alliance Between Charles Sturt University and NSW Department of Primary Industries), Charles Sturt University, Wagga Wagga, NSW, Australia; ^2^School of Animal and Veterinary Sciences, Charles Sturt University, Wagga Wagga, NSW, Australia; ^3^Faculty of Science, Sydney School of Veterinary Science, University of Sydney, Camden, NSW, Australia

**Keywords:** smallholders, animal health management, passive surveillance, Australia, disease reporting

## Abstract

The risks posed for disease introduction and spread are believed to be higher for smallholder livestock producers than commercial producers. Possible reasons for this is the notion that smallholders do not implement appropriate animal health management practices and are not part of traditional livestock communication networks. These factors contribute to the effectiveness of passive disease surveillance systems. A cross-sectional study, using a postal survey (*n* = 1,140) and group interviews (28 participants in three groups), was conducted to understand the animal health management and communication practices of smallholders keeping sheep, cattle, pigs, dairy goats and alpacas in Australia. These practices are crucial for an effective passive surveillance system. Findings indicate that there is a need for improvement in animal health management practices, such as contact with veterinarians and attitudes toward reporting. Results also indicate that these practices differ depending on the livestock species kept, with sheep ownership being associated with lower engagement with surveillance activities and smallholders keeping dairy goats and alpacas having in general better practices. Other factors associated with surveillance practices among participant smallholders are gender and years of experience raising livestock. Despite the differences observed, over 80% of all smallholders actively seek information on the health of their livestock, with private veterinarians considered to be a trusted source. Emergency animal diseases are not a priority among smallholders, however they are concerned about the health of their animals. The finding that veterinarians were identified by producers to be the first point of contact in the event of unusual signs of disease, strengthens the argument that private veterinarians play a vital role in improving passive surveillance. Other producers are also a point of contact for animal health advice, with government agencies less likely to be contacted. The effectiveness of on-farm passive surveillance could be enhanced by developing strategies involving both private veterinarians and producers as key stakeholders, which aim to improve awareness of disease and disease reporting responsibilities.

## Introduction

Biosecurity and animal health management practices of smallholder livestock producers are often perceived as posing an increased risk for disease introduction and spread (1, 2). Key components and drivers of these practices are awareness and knowledge of diseases and attitudes toward monitoring disease and reporting to private veterinarians or relevant authorities. The effectiveness of passive surveillance systems for early detection of disease introductions rely on these practices. Previous studies investigating the effectiveness of surveillance systems in Australia for early detection of foot-and-mouth disease (FMD), reported that improving producer recognition of the presence of unusual signs of disease and reporting these signs would be the most effective strategy for reducing the time from incursion to detection and as such minimizing the potential impact of an FMD outbreak (3, 4).

Whilst increasing producer knowledge and understanding of disease is an essential component of passive surveillance, it is not the only factor that needs to be considered. The actions of an individual are influenced by a number of factors including knowledge, beliefs, attitudes and intentions (5). Studies conducted in the United Kingdom have reflected a disparity between what producers consider to be the “usefulness” of biosecurity practices and the actual implementation of such practices at a farm level, suggesting that the relationship between attitudes and actions in this area requires further investigation (6). Similarly, a study of Danish dairy cattle farmers, reports that despite Danish legislation that farmers with herds larger than 330 per year must develop a farm-specific biosecurity plan, the mandatory plan had not been developed by any of the farmers participating in the study. The researchers speculated that factors that influenced this lack of implementation were a lack of trust in other farmers' ability to maintain adequate biosecurity, uncertainty as to whether other farmers would contribute to the common good, a perception that the risk of disease introduction was low and, the expectation that there would be no social consequences associated with non-compliance (7).

The notion of responsibility is also important to consider. In a study investigating the limitations and incentives in reporting suspicion of Classical Swine Fever amongst pig farmers in the Netherlands, a gap was identified between the expectation of authorities and what pig farmers and veterinary practitioners considered to be their responsibility (8). Strengthening partnerships between government, producers, veterinarians and industry are the main tenets of a biosecurity system based on shared responsibility (9), with such relationships vital to safeguarding livestock industries. Perceptions of risk, consequences, intrinsic and extrinsic benefit or cost, responsibility and social elements, therefore must be at the forefront of discussions on drivers of producer-led passive surveillance.

Whilst the drivers for the on-farm practices of commercial producers are likely to be closely aligned with financial factors, the smallholder sector of livestock producers is arguably more complex. The smallholder sector encompasses a broad range of livestock keepers, in relation to species and number of animals kept, land size, and motivations for keeping livestock (10, 11).

Recent studies focusing on pig producers in Australia have found that herd size and the severity of perceived impact of the disease influence attitudes toward disease reporting. In addition, it was found that recent contact with veterinarians and the keeping of animal health records was less likely in small scale producers (12–14). Studies have also shown that the communication networks that exist between smallholders and industry and government stakeholders are often inadequate (10). It has been suggested that the risk of disease introduction and spread could potentially be reduced through improvements in extension and communication networks, given that this would increase producers' active engagement and participation within their industry (15).

In summary, a limited number of studies have investigated smallholder animal health management and communication practices which define producers' abilities to recognize and report diseases and therefore the effectiveness of passive surveillance systems in the country. This study aims to understand these practices and their influences among smallholder livestock producers in Australia.

## Materials and Methods

The study was conducted in two different phases over a 3 year period from 2013. The first phase involved a cross-sectional survey of Australian smallholder livestock producers (Phase 1) and the second phase involved group interviews with a cohort of these producers (Phase 2). Phase 1 of this study also involved consultation with stakeholders to conduct a stakeholder analysis as reported by Hayes et al. (11). Research proposals for both phases of the study were approved by the Human Research Ethics Committee at Charles Sturt University (20th May 2013) (protocol number 416/2013/05); 9th December 2014 (protocol number 400/2014/52) and 10th November 2015 (protocol number 400/2015/38). For the purpose of this study, smallholders included were those keeping ≤50 head of: (1) cattle and sheep, (2) pigs, (3) dairy goats, and (4) alpacas, noting that participants could keep mixed herds provided that no individual species exceeded 50 head. Smallholders in the first category were those keeping cattle only, sheep only or cattle and sheep.

### Phase 1: Cross-Sectional Study on Smallholder Producers in Australia

#### Development of the Questionnaire

A questionnaire was developed to gather information on demographics and general husbandry (9 questions), biosecurity (17 questions), animal health management (8 questions) and communication networks (3 questions) of smallholders. The questionnaire comprised short-closed, semi-closed and open questions and was prepared for both paper and electronic completion. The questionnaire was piloted with three representatives from state government departments of agriculture and three smallholders, with suggestions incorporated where appropriate. The electronic version of the questionnaire was delivered using the online survey tool, SurveyMonkey®.

#### Questionnaire Distribution

Stakeholders, as identified in the stakeholder analysis conducted as part of Phase 1 of the study (11), who agreed to assist with the cross-sectional study, were contacted to discuss best approaches to the questionnaire distribution. These stakeholders were broadly categorized as state based government departments/authorities, Natural Resource Management (NRM) groups, Catchment Management Authorities (CMA), Landcare Networks (LC), industry associations and community groups. A national level standard approach to distribution of the questionnaire was not possible due to differences between states of Australia in relation to available registers for smallholders and the information available on these smallholders, such the species kept. As such, different approaches of distribution were used for each state and livestock species.

For smallholder producers of cattle and sheep, available sampling frames were state government registers with postal addresses (Qld, SA, Tas and Vic) and with email addresses (WA and NSW). The availability of project funds limited the number of questionnaires which could be delivered by post so a sample of smallholder producers on the government register in Qld, SA, Tas and Vic was selected. The sample size calculation was performed using Epitools epidemiological calculators (epitools.ausvet.com.au), and assumed a population of smallholders >1,000, a 95% confidence level and 5% precision, with ~20–30% of smallholders conducting a specific animal health management practice. The required sample size was between 245 and 322.

The questionnaire was mailed to a randomly selected sample of smallholders from Queensland (*n* = 700), South Australia (*n* = 700), Tasmania (*n* = 696), and Victoria (*n* = 699). For smallholders keeping cattle and sheep in Western Australia and New South Wales the questionnaire was emailed to all government registered smallholders with email addresses (*n* = 780 and *n* = 1,239, respectively). Although availability of resources was a driver for the postal distribution of the survey, a total of 4,814 (2,795 postal, 2,018 electronic distribution) smallholders keeping cattle and sheep were surveyed, which was considered adequate for obtaining a representative sample among this type of smallholder.

For smallholders keeping pigs, the sampling frame was all smallholders identified by Australian Pork Limited (the pork industry representative body) with an available email address (*n* = 897). The questionnaire was distributed by email through Australian Pork Limited. For smallholders keeping alpaca, the survey was distributed through the Australian Alpaca Association, among all members with an available email (*n* = 1,370). For dairy goat smallholders, all producers listed in the publicly available Dairy Goat Society of Australia herd book were sent the questionnaire via post (*n* = 476). Overall, a total of 3,271 questionnaires were distributed by post and 4,286 by email.

The postal questionnaire was sent to the selected smallholders and included the participant information sheet and an addressed, reply paid return envelope. Invitations to complete the questionnaire on-line were sent directly from the assisting organizations in an email containing an introduction to the project and a link to the full information statement and online questionnaire.

A repeat mail-out/electronic contact was used for the distribution of the questionnaire to increase response rate. To reduce potential for non-response bias and encourage participation, an incentive of entry into a lucky draw for five gift vouchers (each of AU $50) for each livestock species group was offered.

#### Data Analysis

Data from the returned questionnaires were entered into Microsoft Excel (2007) and checked for data entry errors. Descriptive and statistical analysis were conducted using IBM SPSS Statistics for Windows, Version 20.0. Armonk, NY: IBM Corp. Associations were investigated between explanatory variables and animal health management practices (dependent variables), using logistic regression analyses. Animal health management practices included in the analysis were: (1) the frequency of livestock inspection; (2) keeping records of animal health events; (3) contact with veterinarians; (4) actions in response to recognizing unusual signs of disease; and (5) sources of animal health information. All of these dependent variables were binary. Association of animal health practices with the explanatory variable “species” was initially investigated using univariable logistic regression analysis. This step was conducted to identify differences in animal health practices between smallholders keeping different livestock species. The next step in the analysis of the data was to investigate those factors (explanatory variables) associated with animal health practices within smallholders keeping the same livestock species. For these analyses, univariable logistic regression was initially conducted to investigate preliminary associations of the animal health practices with a group of explanatory variables. These explanatory variables were: Age, gender, state, property size (hectares), years owning livestock, species kept, number of animals kept and biosecurity knowledge. Variables with *P* < 0.2 in the univariable analysis were investigated further in a multivariable logistic regression model. Prior to building the multivariable model, correlation between these explanatory variables was tested by a chi-square test and only one of a pair of highly correlated variables was considered for inclusion in the multivariable model. Correlations were found between species kept and number of animals kept; property size and number of cattle or sheep kept; and, state and property size. Age and gender were included in the multivariable models as potential confounders. A backward selection method was used to build the multivariable logistic regression model for each animal health practice, with only those explanatory variables with a *P*-value < 0.05 being retained in the final model. Further, first order interaction terms were included to the final models and retained if significant at *P* < 0.05. The model fit was assessed using the Nagelkerke *R*^2^.

To investigate the biosecurity knowledge of smallholders, participants were asked with an open-ended question, to provide a definition of the term, biosecurity. This information was qualitatively analyzed using content analysis and classification of answers into four categories: (0) No knowledge (I don't know/incorrect reference to introduction and spread of diseases); (1) Low level of understanding of biosecurity (general mention of disease prevention but no reference to introduction and/or spread); (2) Moderate level of understanding of biosecurity (correct reference to practices preventing the introduction or the spread); and (3) High level of understanding of biosecurity (correct reference to practices preventing the introduction and spread of disease).

The geographic location of participants in the study (respondents to the survey) according to species kept was mapped by postcode using ArcGISTM 9.3 (ESRI Inc., Redland, CA, USA).

### Phase 2: Group Interviews

#### Recruitment of Participants

Group interviews were undertaken to gain a broader and deeper level of understanding of the attitudes, behaviors and communication networks of smallholders in relation to biosecurity and the management of animal health. This activity provided a follow up to the questionnaire, focussing on areas considered by the research team to be of high interest. The planned structure for the group followed that described by Morgan (16), with a highly moderated structured interview, 8–10 participants per group and three groups. From identified regions of smallholder population in South-Eastern Australia (17–19), three representative areas were selected for inclusion—Riverina region (NSW), South Coast region (NSW), and Euroa/Benalla region (Victoria). In the South Coast and Euroa/Benalla regions, a randomly selected group of 60 smallholders fitting the study criteria were invited to participate in the group interviews, through invitations sent by the NSW Small Farm Networks and Agriculture Victoria, respectively. In the Riverina region, invitations were distributed via community groups, university internal communications, letterbox drops and media. As a result of this recruitment process, eight to ten smallholder producers in each region volunteered to participate in the study. Each participant was offered an AU$50 gift voucher, the provision of lunch and an information package on biosecurity and animal health management.

#### Data Collection and Analysis

Smallholders who made contact with the researchers were emailed a participant information statement and consent form and asked to confirm their willingness to participate via telephone or email. Smallholders confirming their intent to participate were contacted 7 days prior to the group interview, serving as a reminder. Each group interview, which had 2–3 h duration, was facilitated by two researchers, alternating as moderator and scribe. The group interviews comprised structured activities and open discussions in relation to diseases of importance and communication networks. All discussions were recorded via a tape-recorder for subsequent transcription. Descriptive and categorical data from the structured activities was recorded and analyzed in Microsoft® Excel (Windows XP, 2006) and qualitative data was analyzed using applied thematic content analysis (20–22). This approach of analyzing qualitative data allows for the identification and examination of themes using a transparent method. To ensure integrity of the constructs resulting from the thematic analysis two researchers (MH-J and LH) conducted this analysis independently. Qualitative data was read by each researcher and the information coded. A second read was used to validate the initial coding, and from the codes, themes were identified based on topics and frequency of occurrence of these topics.

## Results

### Demographic and Husbandry Characteristics of Smallholders

**Figure 1:**
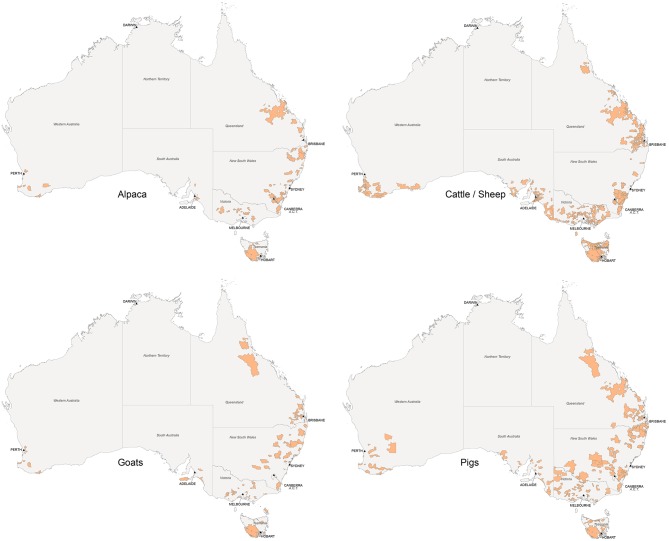
Location by postcode of smallholders participating in a cross-sectional study in Australia in 2013–2015, according to species kept.

A total of 1,140 usable questionnaires were received, including 746 from cattle and sheep smallholders, 198 from pig smallholders, 103 from dairy goat smallholders and 93 from alpaca smallholders. Respondents who did not provide information on number of animals kept, kept no livestock, indicated higher than 50 animals in any of the livestock categories or were otherwise not in the target population have been excluded from the analysis. The response rate, considering only the usable responses, was 14.7, 23.9, 21.6, and 6.8%, for cattle/sheep, pig, dairy goat, and alpaca smallholders, respectively. The distribution of smallholders responding to the survey by species was mapped by postcode and is shown in Figure 1.

Table 1 provides a description of the main demographic and husbandry characteristics of smallholders. Overall, most smallholders were over 45 years of age, the majority of cattle and sheep and pig smallholders being males and for alpaca and dairy goat producers, the majority being female. The distribution of the number of years smallholders kept livestock was different between smallholder types (*P* < 0.05), with cattle and sheep smallholders having kept livestock for longer than other type of smallholders. The majority of participants kept livestock for reasons other than primary income; mainly for extra income, home consumption and as a hobby.

**Table 1:** Demographic and husbandry characteristics of 1,140 smallholder livestock producers participating in a cross-sectional study in Australia in 2013–2015.

**Characteristic**	**Species**							
	**Cattle/sheep**	**%**	**Pigs**	**%**	**Dairy goats**	**%**	**Alpaca**	**%**
**AGE**								
18–24 y	1	0.1	2	1.0	1	1.0	2	2.3
25–34 y	17	2.3	18	9.2	7	7.2	2	2.3
35–44 y	110	15.0	51	26.0	16	16.5	4	4.6
45–54 y	199	27.1	69	35.2	20	20.6	26	30.2
55–64 y	238	32.5	39	19.9	35	36.1	35	40.7
+65 y	168	22.9	17	8.7	18	18.6	17	19.8
**SEX**								
Male	486	67.5^a^	130	68.1^a^	21	21.9^b^	23	26.7^b^
Female	234	32.5	61	31.9	75	78.1	63	73.3
**YEARS KEEPING THE LIVESTOCK SPECIES**								
1–5 y	118	16.3^a^	97	50.0^b^	26	25.2^c^	25	27.2^c^
6–15 y	189	26.1^a^	47	24.2^a^	31	30.1^a^	51	55.4^b^
16–29 y	144	19.9^a^	15	7.7^b^	21	20.4^a^	16	17.4^a^
>30	274	37.8^a^	35	18.0^b^	25	24.3^b^	–	–
**PROPERTY SIZE (HA)**								
<10	223	30.6^a^	30	15.2^b^	49	48.0^c^	30	32.2^a^
10–29	226	31.0^a^	38	19.3^b^	26	25.5^a^^b^	28	30.1^a^
30–79	224	30.7^a^	35	17.8^b^	15	14.7^b^	22	23.7^a^^b^
≥80	56	7.7^a^	94	47.7^b^	12	11.8^a^	13	14.0^a^
**OTHER AGRICULTURAL ACTIVITIES ON PROPERTY**	324	43.4^a^	132	66.7^b^	52	50.5^a^	41	44.1^a^
**STATE**								
New South Wales/ACT	125	16.8	65	32.8	28	27.2	38	41.3
Victoria	148	19.8	47	23.7	25	24.3	18	19.6
Tasmania	126	16.9	18	9.1	8	7.8	9	9.8
South Australia	151	20.2	16	8.1	5	4.9	8	8.7
Western Australia	96	12.9	14	7.1	14	13.6	10	10.9
Queensland	100	13.4	37	18.7	23	22.3	9	9.8
**REASONS FOR KEEPING THE LIVESTOCK SPECIES** ***(multiple responses)***								
Primary income	34	3.3	18	4.9	9	6.5	2	1.7
Extra income	461	44.8^a^	91	24.8^b^	26	18.7^b^	49	41.2^a^
Hobby/family tradition	106	10.3^a^	95	25.9^b^	69	49.6^c^	57	47.9^c^
Home consumption	356	34.6^a^	102	27.8^a^	22	15.8^b^	–	
Pet	106	10.3	15	4.1	13	9.4	11	9.2
Rare breeds	–	–	46	12.5	–	–	–	–

a, b, c *For each row, different superscripts differ P < 0.05*.

In relation to the second phase of this study, a total of 28 smallholder producers participated across three group interviews. The demographic characteristics of group interview smallholders were similar to those of survey participants. Participants kept different livestock species, with most keeping cattle (*n* = 19) and approximately half keeping sheep on their property (*n* = 13). In addition, some kept goats (*n* = 7), horses (*n* = 6), poultry (*n* = 4), and pigs (*n* = 2). Twelve participants only kept one livestock species (cattle, *n* = 6; sheep, *n* = 6), whilst the rest of smallholders kept more than one livestock species. Over 60% of participants were over 45 years of age and the median years of experience raising livestock was 17 years, with a range from 2 to 60 years. Their properties ranged from 5 to 200 ha with a median size of 70 ha. Secondary income (*n* = 15) and family tradition (*n* = 14) were the most common reasons for keeping livestock, followed by home consumption (*n* = 6).

### Animal Health Management Practices

Participant animal health management practices, related to producers' engagement with disease surveillance activities, are shown in Table 2. Most of these practices differed depending on the livestock species kept, with those smallholders keeping dairy goats and alpacas having in general better practices than other smallholders. Smallholders keeping dairy goats and alpacas are more likely (*P* < 0.05) to monitor their animals daily, keep animal health records and have more regular contact with veterinarians than smallholders keeping other livestock species. Furthermore, in relation to contact with veterinarians, results from this study suggest that a proportion of cattle (16.9%) and sheep (27.9%) smallholders had never contacted a veterinarian.

**Table 2:** Animal health management practices of 1,140 smallholder livestock producers participating in a cross-sectional study in Australia in 2013–2015.

**Practice**	**Species**							
	**Cattle/sheep**	**%**	**Pigs**	**%**	**Dairy goats**	**%**	**Alpacas**	**%**
**FREQUENCY OF LIVESTOCK INSPECTION**								
Daily	Cattle 375	62.3^a^	190	97.4^b^	96	97.0^b^	72	81.8^b^
	Sheep 184	66.9^a^						
Weekly	Cattle 200	33.2^a^	4	2.1^b^	3	3.0^b^	12	13.6^b^
	Sheep 76	27.6^a^						
Fortnightly	Cattle 21	3.5	1	0.5	0	–	2	2.3
	Sheep 6	2.2						
Monthly	Cattle 6	1.0	0	–	0	–	2	2.3
	Sheep 9	3.3						
**KEEP RECORDS OF**								
Animals with disease	351	54.8^a^	75	45.2^a^	83	89.2^b^	76	88.4^b^
Animals that died or euthanased	416	63.5^a^	91	54.8^a^	93	96.9^b^	78	90.7^b^
Treatment routine	425	66.3^a^	114	68.7^a^	76	77.6^a^	80	93.0^b^
**CONTACTED A VETERINARIAN IN THE LAST 12 MONTHS**	257	37.4^a^	64	35.8^a^	72	72.7^b^	70	79.5^b^
**If no, last time veterinarian contacted**								
Never	116	16.9^a^^b^	50	27.9^a^	4	4.0^b^	6	6.8^a^^b^
1–2 y ago	119	17.3	34	19.0	12	12.	11	12.5
3–5 y ago	108	15.7^a^	16	8.9^a^	10	10.1^a^	1	1.1^b^
>5 y ago	87	12.7^a^	15	8.4^a^	1	1.0^b^	0	–
**ACTION AFTER IDENTIFYING SYMPTOMS OF DISEASE OR UNUSUAL BEHAVIOR IN YOUR LIVESTOCK** ***(always and sometimes)***								
Do nothing	184	24.7^a^	49	24.7^a^	4	6.3^b^	1	1.7^b^
Treat myself	551	73.8^a^	153	77.3^a^	75	91.5^b^	65	84.4^b^
Call other producer	496	66.5^a^	105	53.0^b^	46	67.6^a^	53	80.3^c^
Call veterinarian	658	88.2	179	90.4	90	94.7	80	97.6
Call livestock agent/saleyard	157	21.0^a^	24	12.1^b^	2	3.4^c^	3	5.6^c^
Call Government agency	269	36.1^a^	87	43.9^a^	22	34.4^a^	14	24.6^b^
Call the Emergency Animal Disease Watch Hotline	158	21.2^a^	61	30.8^a^	0	–	1	1.1^b^
**SEEK INFORMATION ON MANAGEMENT AND HEALTH OF LIVESTOCK**	597	82.9	163	82.3	89	92.7	82	95.3
**MOST USEFUL SOURCES OF INFORMATION**								
Government	311	52.1^a^	98	60.1^a^	36	37.5^b^	31	36.0^b^
Veterinarian	402	67.3^a^	109	66.9^a^	76	79.2^b^	70	46.5^c^
Rural supplier	227	38.0^a^	44	27.0^b^	28	29.2^b^	21	24.4^b^
Other producers	149	25.0^a^	32	19.6^a^	62	64.6^b^	67	77.9^b^
Industry breed groups	72	12.1^a^	34	20.9^a^	41	42.7^b^	34	39.5^b^

a, b, c *For each row, different superscripts differ P < 0.05*.

Despite the differences identified among smallholders and the low veterinary contact reported by some groups of smallholders, most respondents indicated that they would contact a private veterinarian if they saw unusual signs of illness or disease. However, most smallholders also reported that they would treat the animals themselves when faced with unusual signs of disease, and a quarter of smallholders keeping cattle, sheep and pigs reported that they would do nothing in such an event. Other frequent actions reported when faced with unusual signs of disease, were contacting other producers followed by contacting government agencies.

Given the differences in animal health management practices identified between smallholders keeping different livestock species, investigation of other factors influencing these practices was carried out for each type of smallholder, and results are presented in the following sections Animal Health Management Practices of Smallholders Keeping Cattle and Sheep, Animal Health Management Practices of Smallholders Keeping Pigs, and Animal Health Management Practices of Smallholders Keeping Dairy Goats and Alpacas.

The group interviews further investigated animal health management practices, diseases of concern, veterinary contact and actions when faced with unusual signs of disease in their animals, among participant smallholders. A clear animal health management theme emerging from the group interviews was the engagement of smallholders in preventative health measures, including specific preventative treatments, such as vaccination and internal parasite control, and close and frequent monitoring of their animals, as the following quote suggests.

“*I keep a close eye on my animals… I'm constantly around my cattle, so it is unlikely that I will miss any disease*” (Riverina region NSW, smallholder 5)

Participants were asked to list the three diseases considered being of most importance to themselves or their livestock operations and results indicate that producers are mainly concerned about endemic diseases, with internal parasites and clostridial diseases being the most frequently listed diseases. Some emergency and exotic animal diseases, such as foot-and-mouth disease, were listed by some producers; however, these were not considered a priority for smallholders. The thematic analysis of the open discussions identified two major reasons for a disease being considered of concern, these being animal welfare and loss of income, with approximately a third of smallholders identifying each of these as the main reason. Other reasons were the impact on livestock industries and the Australian economy, with 16.1% of smallholders identifying this as a reason, and impact on neighbors (7.7%) and personal/family health (4.8%).

Approximately half of group interview participants (*n* = 13) indicated that they had contacted a veterinarian in the past year. Thematic analysis of the data identified that the two most common reasons for using a veterinarian among participants were for pregnancy testing and animal health problems that producers could not deal with themselves. Other less common reasons for using a veterinarian were vaccinations of companion animals kept on farm and general animal health advice. The quote below provides an example of when smallholders would use a veterinarian.

“(I would use a veterinarian for])… *anything I can't handle myself* ” (South Coast region NSW, smallholder 1)

The group interviews also identified the main barriers or challenges for a more frequent use of veterinarians, with the cost involved with the veterinary services being the main barrier. In agreement with the survey results, although the use of veterinarians could be improved, when participants were asked about the action they would take if faced with unusual signs of disease, most would contact a private veterinarian. However, over half of participants indicated that first they were likely to contact a neighbor, due to the perceived expertise and level of trust, as the following quote indicates.

“*Their experience* (neighbors) *is valuable and can be contacted at any time for opinion and advice*” (Riverina region NSW, smallholder 3)

Only some smallholders would also contact the government veterinarian, with a smallholder showing a lack of trust of some government veterinarians, as seen in the quote below, and another participant perceiving contacting the government veterinarian for unusual signs of disease an overreaction. Most smallholders (*n* = 22) have not heard about the Emergency Animal Disease Watch Hotline.

“*I know that they* (government veterinarians) *don't know what I want to know*” (South Coast region NSW, smallholder 2)

#### Animal Health Management Practices of Smallholders Keeping Cattle and Sheep

Tables 3 and 4 present results of logistic regression analyses investigating associations between demographic and husbandry characteristics and biosecurity knowledge (explanatory variables) and animal health management practices of smallholders keeping cattle and sheep.

**Table 3:** Results of a multivariable logistic regression analysis investigating animal health management practices (contact with veterinarians and record keeping as dependent variables) of 746 smallholders keeping cattle and sheep participating in a cross-sectional study in Australia in 2013–2015 (Only significant associations are shown).

**Practice**	**Producers**	%^*^	***B***	**SE**	**Odds ratio**	**95% CI**	***P***
**CONTACTED A VETERINARIAN IN THE LAST 12 MONTHS**							
Species kept							0.008
Sheep	37	29.8	−0.62	0.25	0.54	0.3–0.9	
Cattle	154	34.6	−0.35	0.33	0.70	0.4–1.4	
Cattle and sheep	60	46.2	0		1.00		
Cattle, sheep, and pigs	6	18.8	−1.84	0.61	0.16	0.1–0.5	
Horses in the property							<0.001
No	144	28.3	0		1.00		
Yes	113	49.1	0.90	0.21	2.45	1.6–3.7	
Biosecurity knowledge							0.006
No-poor	90	31.5	0		1.00		
Mod-High	117	45.2	0.54	0.20	1.72	1.2–2.5	
Property hectares							0.012
<10	70	31.8	−0.39	0.26	0.67	0.4–1.1	
10–29	68	30.8	−0.71	0.24	0.49	0.3–0.8	
30–79	93	41.9	0		1.00		
≥80	20	38.5	0.28	0.41	1.32	0.6–3.0	
**KEEP RECORDS OF ANIMALS WITH DISEASE**							
Gender							0.001
Male	219	51.0	0		1.00		
Female	123	63.1	0.63	0.19	1.87	1.3–2.7	
Species kept							0.002
Sheep	39	35.5	0		1.00		
Cattle	239	60.7	0.89	0.24	2.43	1.5–3.9	
Cattle and sheep	59	53.6	0.63	0.29	1.89	1.1–3.3	
Cattle, sheep, and pigs	14	51.9	0.42	0.48	1.53	0.6–3.9.	
State							<0.001
NSW	84	76.4	0		1.00		
QLD	50	60.2	−0.82	0.33	0.44	0.2–0.8	
SA	52	42.3	−1.43	0.31	0.24	0.1–0.4	
TAS	57	49.1	−1.18	0.31	0.31	0.2–0.6	
VIC	65	51.2	−1.20	0.30	0.30	0.2–0.5	
WA	43	52.4	−1.00	0.33	0.37	0.2–0.7	
**KEEP RECORDS OF ANIMALS THAT DIED OR EUTHANASED?**							
Gender							0.002
Male	261	60.0	0		1.00		
Female	144	71.3	0.62	0.20	1.86	1.3–2.8	
Species kept							0.027
Sheep	48	41.7	0		1.00		
Cattle	270	68.7	0.71	0.24	2.03	1.3–3.3	
Cattle and sheep	80	68.4	0.73	0.31	2.08	1.1–3.8	
Cattle, sheep, and pigs	18	60.0	0.53	0.47	1.70	0.7–4.3	
Property hectares							<0.001
<10	86	45.3	0		1.00		
10–29	127	62.9	0.56	0.22	1.75	1.1–2.7	
30–79	156	77.6	1.20	0.24	3.33	2.1–5.4	
≥80	41	82.0	1.71	0.43	5.54	2.4–12.7	
**KEEP RECORDS OF TREATMENT ROUTINE**							
Biosecurity knowledge							0.016
No-poor	157	63.1	0		1.00		
Mod-High	176	75.5	0.51	0.21	1.67	1.1–2.5	
Property hectares							<0.001
<10	104	55.6	0		1.00		
10–29	133	65.8	0.48	0.26	1.62	1.0–2.7	
30–79	145	75.1	1.01	0.27	2.74	1.6–4.6	
≥80	37	80.4	1.50	0.56	4.49	1.5–13.6	

* *Proportion of producers within each row conducting the specific practice investigated in the model (denominators not provided)*.

**Table 4:** Results of a multivariable logistic regression analysis investigating animal health management practices (monitoring and attitudes toward disease as dependent variables) of 746 smallholders keeping cattle and sheep participating in a cross-sectional study in Australia in 2013–2015 (Only significant associations are shown).

**Practice**	**Producers**	%^*^	***B***	**SE**	**Odds ratio**	**95% CI**	***P***
**HOW OFTEN DO YOU INSPECT YOUR LIVESTOCK? CATTLE (DAILY INSPECTION vs. OTHERS)**							
Gender							0.035
Male	235	59.2	0		1.00		
Female	130	70.3	0.45	0.22	1.57	1.1–2.4	
Property hectares							<0.001
<10	104	72.2	0		1.00		
10–29	128	66.7	−0.23	0.26	0.80	0.5–1.3	
30–79	115	56.1	−0.65	0.25	0.52	0.3–0.8	
≥80	20	40.8	−1.45	0.36	0.23	0.1–0.5	
Horses in the property							0.038
No	401	78.9	0		1.00		
Yes	143	60.3	0.44	0.21	1.55	1.0–2.4	
Years owning livestock							0.011
1–5	41	48.8	0		1.00		
6–15	95	61.7	0.62	0.30	1.85	1.0–3.3	
16–29	69	59.5	0.54	0.32	1.71	0.9–3.2	
≥30	158	68.1	1.00	0.30	2.72	1.5–4.9	
**ACTION AFTER IDENTIFYING UNUSUAL SIGNS OF DISEASE (NEVER CONTACT-GOVERNMENT AGENCY)**							
Property hectares							0.023
<10	89	58.6	0		1.00		
10–29	82	48.8	0.35	0.23	1.42	0.9–2.2	
30–79	67	41.4	0.74	0.24	2.09	1.3–3.4	
≥80	15	40.5	0.52	0.39	1.69	0.8–3.6	
Years keeping livestock							0.016
1–5	40	47.1	0.67	0.29	1.95	1.1–3.5	
6–15	85	59.9	0		1.00		
16–29	45	43.7	0.62	0.27	1.85	1.1–3.2	
≥30	80	43.2	0.73	0.25	2.08	1.3–3.4	

* *Proportion of producers within each row conducting the specific practice investigated in the model (denominators not provided)*.

Species kept (*P* = 0.008) and biosecurity knowledge (*P* = 0.006) were significantly associated with smallholder contact with veterinarians. A higher proportion of producers keeping cattle and sheep (46.2%) had contacted a veterinarian in the last year than smallholders keeping sheep only (29.8%) and those keeping cattle, sheep and pigs (18.8%). In addition, smallholders keeping horses were more likely to have contacted a veterinarian in the past year than those without horses (OR, 2.45; 1.6–3.7; *P* < 0.001). Producers who had a moderate to high understanding of biosecurity were more likely to have contacted a veterinarian (OR, 1.72; 1.2–2.5: *P* = 0.006).

Keeping records of animal health practices was associated with participant gender, property size and species kept (*P* < 0.05; Table 3). In general, keeping these records was more likely among female participants and those with larger properties, and less likely among those smallholders keeping sheep only.

Frequency of animal monitoring and inspection was associated with participant gender, years of experience raising cattle and property size (Table 4). Female respondents and those with more years of experience, reported to inspect their animals more frequently than male and less experienced respondents. In addition, the bigger the property the less frequent the inspection of animals was (*P* < 0.001).

#### Animal Health Management Practices of Smallholders Keeping Pigs

Table 5 presents the results of the logistic regression analyses investigating associations between demographic and husbandry characteristics and biosecurity knowledge (explanatory variables) with animal health management practices among smallholder livestock producers keeping pigs. The main characteristics associated with animal health management practices among smallholders keeping pigs were participant gender, species of livestock kept and the years of experience raising pigs.

**Table 5:** Results of a multivariable logistic regression analysis investigating animal health management related practices of 198 smallholders keeping pigs participating in a cross-sectional study in Australia in 2013–2015 (Only significant associations are shown).

	**Producers**	%^*^	***B***	**SE**	**Odds ratio**	**95% CI**	***P***
**CONTACTED A VETERINARIAN IN THE LAST 12 MONTHS**							
Gender							0.037
Male	34	26.4	0		1.00		
Female	27	45.0	0.75	0.36	2.12	1.1–4.3	
Sows							0.002
0–10	35	26.1	0		1.00		
>10	29	46.8	1.15	0.37	3.15	1.5–6.5	
Sheep							0.002
No	30	42.3	0		1.00		
Yes	34	27.2	−1.08	0.36	0.34	1.2–0.7	
**KEEP RECORDS OF ANIMALS WITH DISEASE**							
**Years keeping livestock**							0.024
1–5	54	65.9	0		1.00		
6–15	18	43.9	−1.07	0.43	0.34	0.1–0.8	
16–29	6	50.0	−0.97	0.69	0.38	0.1–1.5	
>30	11	37.9	−1.23	0.53	0.29	0.1–0.8	
**KEEP RECORDS OF ANIMALS THAT DIED OR EUTHANASED?**							
Age							0.02
18–34	13	72.2	1.27	0.62	3.56	1.1–12.0	
35–44	21	43.8	0		1.00		
45–54	38	63.3	1.03	0.43	2.81	1.2–6.5	
55–64	24	72.7	1.47	0.52	4.35	1.6–12.1	
>65	4	40.0	0.06	0.75	1.06	0.2–4.6	
Sheep							0.01
No	47	72.3	0		1.00		
Yes	55	51.9	−0.93	0.36	0.39	0.2–0.8	
**KEEP RECORDS OF TREATMENT ROUTINE**							
Sheep							0.033
No	49	77.8	0		1.00		
Yes	65	63.1	−0.82	0.39	0.44	0.2–0.9	
**ACTION AFTER IDENTIFYING UNUSUAL SIGNS OF DISEASE (NEVER CONTACT-NEIGHBOR/FRIEND/OTHER PRODUCER)**							
Years							0.017
1–5	62	83.8	0		1.00		
6–15	24	64.9	−1.00	0.50	0.37	0.1–1.0	
16–29	41	36.4	−2.07	0.73	0.13	0.0–0.5	
>30	4	56.0	−1.01	0.56	0.36	0.1–1.1	

* *Proportion of producers within each row conducting the specific practice investigated in the model (denominators not provided)*.

Veterinary contact in the past 12 months was more likely among female pig smallholders (*P* = 0.037), smallholders keeping more than 10 sows *(P* = 0.002) and those not keeping sheep in their property (*P* = 0.002). Keeping animal health records was more likely (*P* < 0.05) among younger and less experienced smallholders and those with no sheep on their property.

#### Animal Health Management Practices of Smallholders Keeping Dairy Goats and Alpacas

Limited significant associations were found during the logistic regression analyses for smallholders keeping dairy goats. In agreement with results reported for the previous groups of smallholders, univariable analysis showed that dairy goat smallholders keeping sheep were less likely to have contact with veterinarians than those not keeping sheep (*P* = 0.04); however, when age and gender were included in the multivariable analysis as confounders, there was no association between keeping sheep and contact with veterinarians. The only practice where significant associations were identified by the multivariable logistic regression analysis was contacting a government veterinarian in the event of unusual signs of disease or behavior in goats (data not shown). Smallholders with more years of experience raising dairy goats were more likely (*P* = 0.04) to contact a government veterinarian than less experienced smallholders. No significant associations were observed between alpaca smallholder demographics and their animal health management practices.

### Information Sources on Livestock Management and Health

The vast majority of smallholders indicated that they sought information on the management and health of their livestock (Table 2). Veterinarians were considered to be the most useful source of information by all smallholders (>65%), with the exception of alpaca smallholders, of which less than half of respondents considered veterinarians as a useful source of information. When comparing other sources of information among the different groups of smallholders, dairy goat and alpaca smallholders were more likely (*P* < 0.05) to consider information from other producers and industry breed groups useful, and cattle and sheep and pig producers were more likely (*P* < 0.05) to consider government a useful source of information.

Within smallholders keeping cattle and sheep, and those keeping pigs, some significant associations were observed between the reported information seeking behavior and some explanatory variables. For smallholders keeping cattle and sheep, female and younger producers were more likely to seek information on livestock management and health than male (*P* = 0.028) and older producers (*P* < 0.001). Regarding usefulness of different sources of information, producers from NSW (54.9%) and Western Australia (59.5%) were more likely (*P* < 0.001) to consider government agencies as useful sources than producers from other states. Results also suggest that those smallholders keeping only sheep are less likely to find veterinarians a useful source of information than other smallholders within this group (*P* = 0.006), with aligns with the frequency of veterinary used previously reported.

Within smallholders keeping pigs, those with less experience raising pigs and with <10 sows, were more likely (*P* < 0.001) to seek information on pig management and health. Over 90% of producers with <5 years of experience reported seeking information compared to 68.6% among producers with more than 30 years of experience. In addition, less experienced smallholders were more likely (*P* < 0.05) to rely on other producers as useful sources of information.

## Discussion

The aim of this study was to develop an understanding of the demographic and husbandry characteristics that may influence smallholder livestock producer's engagement with animal health management and disease reporting, key practices of the on-farm component of a passive surveillance system. Over a thousand smallholder producers participated in the study; being one of the first studies among this sector of livestock producers in a developed country, with this number of participants. However, one of the limitations of the study was the low response rate, which could be due to the distribution methods and the topic of the study, which are known factors influencing response rate (23). Given no specific registers for smallholder producers exist and distribution was done through other organizations due to confidentially reasons, follow-up with non-respondents was not possible and as such, selection bias could not be assessed. A selection bias is acknowledged in the group interviews with the possibility that those who agree to take part in research of this nature, may already possess an interest in the subject matter and as such, may not be representative of the smallholder population. Although participants represented a diversity of smallholders in relation to location and key demographic and farm characteristics results need to be interpreted with caution. Another potential limitation of the study is in relation to the data analysis approach, which could be associated with the multiple testing problem. This problem refers to the probability of false positives or Type I errors, which increases when more tests are conducted to investigate the association of a specific explanatory variable with several dependent variables. In this study, a total of 12 dependent variables (animal health management practices) were investigated for each smallholder type, but associations with the explanatory variables were only found for a low number of animal health management practices (up to six). As such, although possible, we believe the multiple testing problem is unlikely to have caused major impacts on the results. When interpreting results from the multivariable logistic regression models is important to consider that these models aim to identify potential factors influencing animal health management practices, and should not be used as a predictive model. Findings from this study, generally indicate that sheep ownership was a factor associated with lower levels of inspection and engagement with veterinarians. To maximize the likelihood of the early detection of disease, it is essential to regularly observe livestock, with delays having potential economic and eradication implications (24). With over 95% of participants in the current study reporting that they inspected their livestock at a minimum weekly interval, it can be argued that smallholders are effectively engaging in passive surveillance. However, whilst this would appear to indicate that early signs of disease could potentially be identified by producers, for diseases that can be spread during the incubation period, such as foot-and-mouth disease, a weekly inspection interval, as reported by a third of smallholders keeping cattle and sheep, would not be adequate. The effectiveness of inspection is also dependent on the level of knowledge of clinical signs of disease and the actions taken once such signs are observed (24, 25). It is also important that accurate animal health records are maintained given that these can help to identify patterns of disease or deaths. With the exception of alpaca producers, improvement in record keeping is clearly needed across all species.

The time between when a problem is recognized and a veterinarian consulted has been shown to be a major influence on time to disease detection (3). In the current study it was found that the majority of smallholders, when observing symptoms of disease, would attempt to treat it themselves. This is likely to be influenced by the degree to which a disease is considered by the individual farmer to be of concern to their operation. The group interviews provided an opportunity for further exploration of some of the questionnaires key areas of interest. Results indicate that endemic diseases are the main diseases considered to be of importance. As reported in similar studies (26), despite smallholders being concerned about the health of their animals, they do not consider EADs to be a priority and as such, the effectiveness of animal inspection for early detection of diseases comes into question. Whilst EADs are understandably a high priority to those involved in protecting the integrity of the Australian livestock industries, for the individual producer it can be a case of competing priorities and motivations. Whilst for cattle and sheep producers in this study the motivation for keeping livestock was primarily associated with obtaining additional income and home consumption, for those keeping other species the motivation was less clear. This highlights the difficulty in making generalizations about smallholders as they are clearly not a homogenous group (11). Studies have shown that animal welfare is a motivator for disease prevention, across all species, particularly for those with non-intensive systems (27). The current study supports these findings, with group interview participants identifying animal welfare the main reason of concern in relation to animal health. For many smallholders, livestock are considered pets, regardless of whether they ultimately end up slaughtered for home consumption (18). These findings are interesting to consider in relation to the previously identified selection bias. If producers who agree to participate in studies of this nature are assumed to be more engaged in the topic of interest, the fact that they do not prioritize EAD's, suggests that more work needs to be done across the whole smallholder sector in terms of biosecurity engagement.

The relationship between producers and veterinarians has been explored in previous studies (11, 15, 28). For producer-led passive surveillance to be effective, producers must trust both, those from whom they receive information and those to whom they provide information (4). Veterinarians are considered to be a trusted stakeholder, thereby placing them in a strong position to influence the behavior of smallholders (11, 15, 29). In the current study, the frequency of contact with the veterinarian was different between species, with a low contact identified among cattle and sheep and pig smallholders. A relationship with a veterinarian is an important aspect of animal ownership and the finding that a proportion of cattle and sheep and pig smallholders had never contacted a veterinarian requires consideration. Whilst the reasons for this were not explored in this study, the level of experience of such producers as compared to alpaca and dairy goats may, in part, explain this finding. Horse ownership was positively associated with veterinary contact, possibly indicating that for those keeping higher value animals, their relationship with a veterinarian is established and as such may cross over to their livestock operation.

Producers with a moderate to high understanding of biosecurity were also more likely to have contacted a veterinarian, suggesting that an understanding of biosecurity may be a positive influence on behavior and attitudes toward surveillance (27). This supports the suggestion that producers who discuss the application of biosecurity measures with veterinarians are more likely to engage in stronger biosecurity behavior (30).

Results in relation to information sources should be interpreted with caution, as the questionnaire distribution method had the potential to impact the outcomes in this component of the survey. The involvement of industry bodies in the distribution of the questionnaire to alpaca and dairy goat producers, meant that the questionnaire was only distributed to those already aligned with an industry organization. Previous studies have reported a considerable level of mistrust of government sources (11, 15, 29, 31), a finding supported by the current study, with almost half of producers indicating that they would never call the State Department of Primary Industries or Agriculture in the event of unusual symptoms. In addition, there were state differences observed with producers from NSW and WA being more likely to contact a government source. At the time that this study was conducted, these states were the only Australian states or territories that had government services tasked solely with supporting smallholders, leading to the suggestion that services such as these, are an effective method of engaging smallholders.

It could be argued that for more experienced producers, in this study those keeping cattle and sheep, past exposure to a higher level of service from government agencies may have resulted in an ongoing positive relationship with such services. In recent decades there has been a reduction in government extension services (3, 15, 32–34) which for those in “newer” industries such as the alpaca industry, may mean that they have had no past experience with government support and as such, may not consider them to be a useful source of information as a result of this. The relationship between producers must not be underestimated when it comes to animal health management, particularly as “other producers” were shown to be one of the primary contacts in the event of the observation of unusual symptoms of disease.

In summary, this study provides an insight into the animal health management practices of smallholder livestock producers in Australia and identifies some influencing characteristics that should be considered when developing strategies for improving their engagement with the surveillance system in the country. Species kept, the level of experience, the location as well as the local networks used by the smallholders are important factors to consider. It is important that the correct health related information is shared between producers, leading the authors to suggest that well-informed “champion” producers could be included as part an overall producer-led passive surveillance strategy. The need for the flow of information from government sources both to and subsequently between experienced and less experienced producers, highlights the importance of understanding and building upon these relationships.

## Data Availability

The datasets for this study will not be made publicly available because All data was collected under confidentiality and following Human Ethics Committee requirements, data was not to be shared beyond the research team.

## Ethics Statement

Research proposals for both phases of the study were approved by the Human Research Ethics Committee at Charles Sturt University (20th May 2013 (protocol number 416/2013/05); 9th December 2014 (protocol number 400/2014/52) and 10th November 2015 (protocol number 400/2015/38).

## Author Contributions

MH-J, LH, and J-AT designed the study and developed the questionnaire and the focus group activities. LH led the distribution of the questionnaire and the recruitment of focus group participants. MH-J and LH analyzed questionnaire data. MH-J, LH, RW, and LR organized and facilitated focus group discussions. MH-J and LH analyzed the focus group data. MH-J and LH prepared the manuscript. J-AT, RW, and LR provided input to the manuscript.

### Conflict of Interest Statement

The authors declare that the research was conducted in the absence of any commercial or financial relationships that could be construed as a potential conflict of interest.
